# Climatology of nocturnal low-level jets over North Africa and implications for modeling mineral dust emission

**DOI:** 10.1002/jgrd.50394

**Published:** 2013-06-21

**Authors:** S Fiedler, K Schepanski, B Heinold, P Knippertz, I Tegen

**Affiliations:** 1 School of Earth and Environment, University of LeedsLeeds, UK; 2 Now at Leibniz Institute for Tropospheric ResearchLeipzig, Germany; 3 Leibniz Institute for Tropospheric ResearchLeipzig, Germany

**Keywords:** nocturnal low-level jet, Harmattan, dust emission, Saharan heat low, ERA-Interim reanalysis, diurnal cycle

## Abstract

[1] This study presents the first climatology for the dust emission amount associated with Nocturnal Low-Level Jets (NLLJs) in North Africa. These wind speed maxima near the top of the nocturnal boundary layer can generate near-surface peak winds due to shear-driven turbulence in the course of the night and the NLLJ breakdown during the following morning. The associated increase in the near-surface wind speed is a driver for mineral dust emission. A new detection algorithm for NLLJs is presented and used for a statistical assessment of NLLJs in 32 years of ERA-Interim reanalysis from the European Centre for Medium-Range Weather Forecasts. NLLJs occur in 29% of the nights in the annual and spatial mean. The NLLJ climatology shows a distinct annual cycle with marked regional differences. Maxima of up to 80% NLLJ frequency are found where low-level baroclinicity and orographic channels cause favorable conditions, e.g., over the Bodélé Depression, Chad, for November–February and along the West Saharan and Mauritanian coast for April–September. Downward mixing of NLLJ momentum to the surface causes 15% of mineral dust emission in the annual and spatial mean and can be associated with up to 60% of the total dust amount in specific areas, e.g., the Bodélé Depression and south of the Hoggar-Tibesti Channel. The sharp diurnal cycle underlines the importance of using wind speed information with high temporal resolution as driving fields for dust emission models.

**Citation:** Fiedler, S., K. Schepanski, B. Heinold, P. Knippertz, and I. Tegen (2013), Climatology of nocturnal low-level jets over North Africa and implications for modeling mineral dust emission, J. Geophys. Res. Atmos., 118, 6100-6121, doi:10.1002/jgrd.50394

## 1 Introduction

[2] Mineral dust constitutes the largest fraction of atmospheric aerosol by mass and plays an important role in the Earth system. Mineral dust aerosols interact with radiation directly, through absorption and scattering of shortwave and longwave radiation [*Haywood et al.*, 2003; *Sokolik and Toon*, 1996], and indirectly, by serving as cloud condensation and ice nuclei that determine cloud optical properties [*Lohmann and Feichter*, 2005; *Karydis et al.*, 2011] and precipitability [*Rosenfeld et al.*, 2001]. In addition to influences on weather and climate, mineral dust has impacts on human health [*Griffin*, 2007] and fertilizes terrestrial [*Okin et al.*, 2004] and marine ecosystems [*Mahowald et al.*, 2005]. The dominant source for dust aerosols on Earth is North Africa [*Tegen and Schepanski*, 2009], from where particulate matter can be transported toward Europe, America, and beyond [*Kaufman et al.*, 2005; *Koren et al.*, 2006; *Ben-Ami et al.*^2009^].

[3] Realistic simulations of mineral dust aerosols depend on the accurate time, location, and amount of emission fluxes. In most state-of-the-art models, dust emission is described as a nonlinear function of surface characteristics and the momentum transfer from the atmosphere to the ground [e.g., *Marticorena and Bergametti*, 1995; *Shao*, 2001]. The erodibility depends on surface characteristics, such as soil grain size, chemical composition, soil moisture, roughness lengths, and vegetation cover [*Tegen and Schepanski*, 2009]. Influences of these source-dependent factors on dust emission are often parametrized by a threshold for the momentum transfer to the surface, expressed in terms of friction velocity or near-surface wind speed [e.g., *Tegen et al.*, 2002; *Ginoux et al.*, 2001]. After exceeding the threshold, the dust emission flux often depends cubic on the downward momentum flux [e.g., *Ginoux et al.*, 2001; *Pérez et al.*, 2011]. This implies that the representation of the wind speed distribution is crucial for simulating dust emission.

[4] Further improvements of simulating the mineral dust budget for weather forecast and climate applications require a systematic analysis of individual parameters relevant for the dust emission flux. The evaluation of both soil parameters in the dust emission parameterization and meteorological mechanisms generating peak winds is important [*Knippertz and Todd*, 2012, and references therein]. Different meteorological processes have been identified as potential generators for dust-emitting peak winds near the surface. The understanding of the relative importance of these mechanisms is, however, incomplete. One of the relevant meteorological processes is the downward mixing of momentum from the Nocturnal Low-Level Jet (NLLJ) [*Washington and Todd*, 2005; *Knippertz*, 2008; *Schepanski et al.*, 2009], a layer of relatively high wind speed in a few hundred meters above the ground during the night. While a frequent occurrence of NLLJs and their contribution to mineral dust emission in North Africa has been suggested qualitatively [*Schepanski et al.*, 2009], a climatological estimate of the phenomenon is missing.

[5] This study presents a climatological assessment of NLLJs and their quantitative contribution to dust emission over North Africa. A new automatic algorithm for detecting NLLJs in atmospheric models has been developed and applied to the European Centre for Medium-Range Weather Forecasts (ECMWF) ERA-Interim reanalysis and forecasts for 1979–2010 for this purpose. An overview of literature on NLLJs is given in section 2, followed by the description of the detection algorithm and the data in section 3. Results from the validation of NLLJs in the ECMWF model are shown in section 4. The climatology of the NLLJ frequency, of the jet characteristics, of the dust emission, and the relative importance of NLLJs for dust emission are presented in sections 5–7. A discussion of the findings and their implication for mineral dust modeling are given in section 8. Section 9 draws conclusions from this work.

## 2 Background on NLLJs

[6] Low-level jets (LLJs) are wind speed maxima in the lower troposphere, as defined in the meteorological terminology data base METEOTERM run by the World Meteorological Organization (http://wmo.multicorpora.net/METEOTERM). Most previous studies focus on LLJs in specific parts of the world, e.g., north-east Australia [*May*, 1995], the Nares Strait channel near Greenland [*Samelson and Barbour*, 2007], the USA [*Bonner*, 1968; *Hoxit*, 1975; *Whiteman et al.*, 1997; *Banta et al.*, 2003], Cabauw in the Netherlands [e.g., *Baas et al.*, 2009], the Persian Gulf [*Giannakopoulou and Toumi*, 2012], the Bodélé Depression in Chad [*Washington and Todd*, 2005; *Washington et al.*, 2006], and the Sahara [*Schepanski et al.*, 2009]. Some work has been done on compiling a global distribution of LLJs. These include the qualitative review by *Stensrud* [1996] and the quantitative investigation of diurnally varying LLJs based on a 21 year reanalysis data set by *Rife et al*. [2010]. The present study focuses on LLJs that occur at night. These NLLJs reside close to the top of the nocturnal surface inversion [*Blackadar*, 1957; *Baas et al.*, 2009; *Gross*, 2012]. Studies for the Great Plain LLJ indicate that the jet is found in variable heights above the surface inversion top at night [*Bonner*, 1968; *Whiteman et al.*^1997^].

### 2.1 Formation of NLLJs

[7] Different meteorological conditions can generate NLLJs. While some mechanisms have been identified for specific seasons and regions of North Africa [*Parker et al*., 2005; *Washington and Todd*, 2005; *Todd et al*., 2008], an assessment of the relative importance of the mechanisms in entire North Africa does not exist. The formation of NLLJs can be explained with the aid of different conceptual approaches.

[8] The classical theory by *Blackadar* [1957] describes the NLLJ formation using the concept of an inertial oscillation, a process tied to a decoupling of the air flow from surface friction. One of the requirements is radiative cooling that stabilizes the surface layer. The associated weaker dynamical friction due to reduced eddy viscosity enables an acceleration of the air aloft, a process primarily occurring over land at night. *Blackadar* [1957] assumes a complete frictional decoupling and a constant horizontal pressure gradient. Under these theoretical conditions, the wind change is solely determined by the Coriolis force. The actual wind then oscillates around the geostrophic wind vector, leading to super-geostrophic speed and preventing a geostrophic adjustment [*Stull*, 1988]. The actual wind speed in the core of the NLLJ is determined by both the initial ageostrophic wind at the time of decoupling and the geostrophic wind [*Blackadar*, 1957]. While the geostrophic wind is driven by horizontal pressure gradients on the mesoscale to synoptic scale, surface friction has a strong influence on the ageostrophic wind component. The oscillation period is a function of the Coriolis parameter *f*, which depends on the geographical latitude. The time of maximum enhancement of the wind speed occurs when the actual wind vector and the geostrophic wind vector are aligned, which is between a quarter and a half of the oscillation period T. Half a period (T/2 = *π*/*f*) corresponds to 12 h at 30°N and 17.5 h at 20°N. Highest wind speeds and, therefore, potentially largest mineral dust emission are expected when the time of maximum enhancement of the NLLJ agrees with the time of the NLLJ breakdown. Even though the inertial oscillation is a simplified concept of a two-dimensional problem over flat terrain under neglection of any frictional effects, it is found to capture observed conditions in the United States reasonably well [*Bonner and Paegle*, 1970]. An even better agreement with the variations of boundary layer winds is found when the diurnal cycles of both the eddy viscosity and the geostrophic wind speed are included [*Bonner and Paegle*, 1970]. A recent revision of the classical inertial oscillation by *Van de Wiel et al*. [2010] considers frictional effects in the nocturnal boundary layer (BL) during the inertial oscillation by replacing the geostrophic wind by a more general equilibrium wind vector [*Van de Wiel et al.*^2010^].

[9] Other NLLJ generating conditions include compensating air flows for low-level baroclinicity in regions of differential heating and cooling. Evidence from the Arabian Peninsula shows that baroclinicity can be a more important forming mechanism for LLJs than the inertial oscillation [*Giannakopoulou and Toumi*, 2012]. In contrast to the concept of an inertial oscillation, LLJ formation due to baroclinic condition can occur during day and night. However, a nocturnal enhancement of the LLJ can nevertheless be expected due to the reduction of the eddy viscosity in the nocturnal BL. For instance, *Stensrud* [1996] documents that the enhancement of LLJs at night can occur over regions with land-sea contrasts. *Grams et al.* [2010] describe the baroclinicly driven inflow of air from the Atlantic over parts of West Africa and show a significant increase of the wind speed in the stably stratified air behind the sea-breeze front at night.

[10] In addition to coastlines, baroclinic conditions may develop over complex terrain. After sunset, the surface and subsequently the air above higher lying terrain cools more than in a valley. In response to the horizontal pressure gradient, a downhill flow develops with a wind speed maximum close to the surface. These circulations over sloping terrain can generate LLJ profiles, a process first documented by *Bleeker and Andre* [1951]. *Schepanski et al.* [2009] suggest that a relatively large number of NLLJs occur over mountainous regions in North Africa, pointing at baroclinicity over complex terrain as a driving mechanism. Previous work further indicates that NLLJs are generated by larger-scale baroclinicity during the West African monsoon in the Sahel in northern hemisphere summer. *Parker et al.* [2005] describe these NLLJs at the southern margins of the Saharan heat low embedded in the monsoon air flow. Observations give evidence for the nocturnal acceleration of these LLJs [*Abdou et al.*, 2010; *Bain et al.*, 2010; *Pospichal et al.*, 2010] with largest core wind speeds in the morning (06–07 UTC at Niamey for 2006) [*Lothon et al.*, 2008].

[11] NLLJ structures also emerge as a dynamical response to orographic channeling [*Samelson and Barbour*, 2007; *Washington and Todd*, 2005; *Washington et al*., 2006; *Todd et al*., 2008; *Schepanski et al*., 2009]. Channeling may also cause LLJ structures throughout the day, but a diurnal cycle of the jet wind speed is nevertheless expected based on observational evidence over the Bodélé Depression, Chad [*Washington et al*., 2006; *Todd et al*., 2008]. The results show a distinct diurnal cycle of the near-surface wind speed under both weak and strong large-scale forcing [*Todd et al*., 2008]. This indicates a diurnal variation in the eddy viscosity, which implies a reduction of frictional effects as conditions for a nocturnal acceleration of the LLJ. The diurnal amplitude of the near-surface wind speed is, herein, smaller under a large horizontal pressure gradient compared to a weaker background forcing. This can be linked to more frequent or more efficient shear-induced turbulence beneath the NLLJ. It is this interruption of the NLLJ development that keeps the NLLJs relatively short-lived under strong background flows. Both the weakening or intermittent erosion of the NLLJ by downward mixing of the momentum and the short time periods between mixing events for recovering from the momentum loss means that the nocturnal enhancement of a NLLJ under strong large-scale forcing is overall smaller. Less net momentum gain of the NLLJ implies that a potential increase in the near-surface wind speed from a sudden downward mixing event in the mid-morning is relatively small. It is this process that keeps the amplitude of the diurnal cycle in both the NLLJ and the near-surface level relatively small when the background flow is strong.

### 2.2 Definition of LLJs

[12] Different definitions for LLJs, NLLJs, and nocturnal jets in previous studies and meteorological glossaries show that there is no universal agreement on the terminology, a problem already raised by *Stensrud* [1996]. For instance, the definition for “nocturnal jets” by METEOTERM (http://wmo.multicorpora.net/METEOTERM) follows the idea of an inertial oscillation proposed by *Blackadar* [1957], while the American Meteorological Society more generally defines it as “Usually, a low-level jet that occurs at night.” (http://amsglossary.allenpress.com/glossary). The term LLJ is, herein, used for a wind speed maximum in the lower part of the troposphere in both glossaries. Previous studies on LLJs, NLLJs, and nocturnal jets can be summarized under this more general term LLJ.

[13] The different definitions of LLJ structures in previous studies is tightly connected to the applied identification methods. The variety of methods range from plain wind speed maximum analysis to more complex, physically motivated, and automated approaches. Each of the different techniques have their own advantages and disadvantages for the specific research interests. A short summary is given in the following.

[14] Nocturnal jets are identified in radiosondes over north-east Australia by finding a wind speed maximum below 1500 m above ground level (agl) that shows an increase of the wind speed over night and decays around sunrise [*May*, 1995]. This approach is tailored toward inertial oscillations. Other studies more generally address NLLJs with more generous identification criteria. For instance, *Banta et al.* [2003] investigate NLLJs in observations averaged over 15 min for 10 nights over Kansas by choosing the lowest wind speed maximum in the vertical profile as the NLLJ. This approach does not consider a critical vertical wind shear above the NLLJ, which is useful for confining the NLLJ to a vertically narrow band as suggested by *Stensrud* [1996]. While the confinement beneath the jet core is naturally given due to the effect of surface friction, a restriction of the vertical extent of the NLLJ above the nose is a useful addition for the identification.

[15] A criterion for the decrease of wind speed above the NLLJ core is applied by a number of previous studies [e.g., *Bonner*, 1968; *Whiteman et al.*, 1997; *Baas et al.*, 2009]. The study by *Bonner* [1968] determines NLLJs in 2 years of radiosondes observations across the United States by using four criterion sets of an absolute core wind speed of at least 12 m s^−1^and a certain wind speed decrease above the LLJ nose. This classification scheme by *Bonner* [1968] has been used later by *Whiteman et al.* [1997] to study LLJs over the USA independent of the time of day. *Baas et al.* [2009] identified NLLJs when the maximum resides below 500 m, is at least 2 m s^−1^, and 25% faster than the following minimum. While this height range works well for typical midlatitude BLs over the Netherlands, observations from Africa show that NLLJs can reside in altitudes exceeding 500 m agl [*Todd et al.*, 2008; *Pospichal et al.*, 2010; *Rife et al.*, 2010].

[16] *Rife et al.* [2010] use 21 years of reanalysis data to compile a global climatology of the “NLLJ index” defined as the wind speed at a fixed height of 500 m agl at midnight that is larger than the wind speed 12 h earlier and than the wind speed at 4000 m. Their spatial distribution of the “NLLJ index” indicates where diurnally varying LLJs are located but does not provide the absolute wind speed and height of the jet core. Further, this approach does not take into account that the synoptic conditions may change in a 12 h period. Reanalysis data have also been used to compile a mean NLLJ climatology over the Bodélé Depression in Chad based on pressure levels by *Washington and Todd*[2005]. *Schepanski et al.* [2009] and *Crouvi et al.* [2012] use wind speed differences between standard pressure levels as indicator for NLLJs for entire North Africa. This approach does not provide the absolute wind speed and height of the NLLJ core and is not applicable over mountains.

[17] The objective of the present study is to identify LLJs that develop at night and potentially lead to mineral dust emission during the following morning (section 2.3). Based on this research aim and following METEOTERM (http://wmo.multicorpora.net/METEOTERM), NLLJs over North Africa are defined in this study as wind speed maxima in the nocturnal BL that form above a stably stratified surface layer and have an appreciable vertical wind shear. This NLLJ definition is a more restrictive form of the relatively general term LLJ. The present study includes but is not limited to jets of super-geostrophic speed, as proposed by *Blackadar*[1957].

### 2.3 Impact on Dust Aerosol

**Figure 1:**
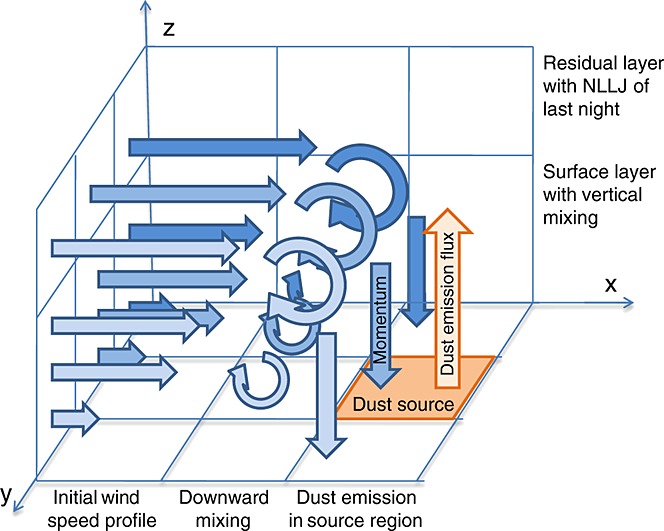
Schematic diagram showing the downward mixing of NLLJ momentum during the morning hours as proposed by the literature. Turbulent mixing transports momentum toward the surface, which leads to dust emission in source areas, when the specific threshold velocity is exceeded. For details, see section 2.3.

[18] The effect of NLLJs on mineral dust aerosol is twofold. On the one hand, NLLJs efficiently transport uplifted dust [*Kalu*, 1979]. On the other hand, the downward mixing of NLLJ momentum by turbulence increases the near-surface wind speed and potentially leads to mineral dust emission in source areas [*Todd et al.*, 2008; *Schepanski et al.*, 2009; *Heinold et al.*, 2011]. This downward mixing of momentum from the NLLJ is schematically depicted in Figure 1. The associated peak winds and dust emission during the mid-morning can be larger than midday values in wide areas across North Africa [*Schepanski et al.*, 2009; *Crouvi et al.*, 2012]. It is interesting that relatively higher near-surface wind speed for 04–07 LT than at 09–15 LT has been documented in an early study over the Sudan, for which theodolite measurements were made twice a day in 1935 and 1936 at Khartoum [*Farquharson*, 1939]. High wind speeds during the mid-morning are further observed at Niamey, Niger, and Nangatchori, Benin [*Lothon et al.*, 2008; *Abdou et al.*^2010^].

[19] The downward mixing of NLLJ momentum has been suggested as an important mechanism for generating near-surface peak winds and dust emission in North Africa during the mid-morning [e.g., *Washington and Todd*, 2005; *Knippertz*, 2008; *Schepanski et al.*, 2009; *Abdou et al.*, 2010; *Marsham et al.*, 2011]. A quantification in terms of dust source activation (DSA) frequency suggests that 65% of the dust emission events in entire North Africa is linked to the morning breakdown of NLLJs, a result which is based on the time of DSA from satellite observations for 2006–2008 [*Schepanski et al.*^2009^]. However, a 20–30 year climatology of the NLLJ breakdown and an estimate of the associated amount of emitted dust have not been quantified yet. These are the aims of the present study.

[20] Turbulence mixes the NLLJ momentum toward the surface (graphically depicted as eddies in Figure 1). *Lothon et al.* [2008] show observational evidence for this downward mixing from West Africa. A turbulent flow is caused by vertical wind shear and by surface heating leading to a reduced static stability [*Lenschow and Stankow*, 1979; *Van de Wiel et al.*, 2003, and references therein]. A useful concept for describing the onset of turbulence in an atmospheric flow, and thereby the timing of downward mixing of momentum from the NLLJ, is the Richardson number. This dimensionless number is defined as the ratio of the static stability and the vertical wind shear. Turbulence occurs when the Richardson number is below a critical threshold, e.g., in a situation of a large vertical wind shear in weakly stable conditions. *Banta et al.* [2003] show that the critical bulk Richardson number beneath a NLLJ over flat terrain describes the onset of turbulent kinetic energy production for values smaller than 0.4. Continuous turbulence is observed for moderately stable situations characterized by Richardson numbers smaller than 0.25–0.3. Intermittent turbulence occurs for very stable situations with Richardson numbers larger than 0.3 [*Banta et al.*^2003^].

## 3 Data and Method

### 3.1 ECMWF ERA-Interim

[21] The basis for the statistical investigation of NLLJs in North Africa is the ERA-Interim data from the European Centre for Medium-Range Weather Forecasts (ECMWF) for 1979–2010 [*Dee et al.*, 2011]. The choice of ERA-Interim for the present work instead of radiosonde observations was based on (1) the sparse observation network, (2) the lack of long-term records over most of North Africa, and (3) too few radiosonde ascents per night. These shortcomings do not enable to capture the nocturnal development of NLLJs by observations in most areas of North Africa, especially in remote regions of the Sahara desert that are particularly interesting from the perspective of dust emission.

[22] The six-hourly reanalysis provides instantaneous fields on a 1°×1° horizontal grid and 60 vertical levels, which are terrain following close to the surface and gradually adjust to pressure coordinates in the free troposphere. The reanalysis of the horizontal wind components, the air temperature, and the specific humidity are used for the NLLJ climatology. Temporally higher resolved data is beneficial for understanding the development and the breakdown of NLLJs as well as the associated mineral dust emission flux. In order to increase the temporal resolution of the diurnal cycle for process studies, three-hourly ERA-Interim forecasts are used. The present study uses the forecasts for +03 to +12 h initialized at 00 UTC and 12 UTC. All variables from ERA-Interim forecasts are instantaneous values, except the 10 m wind gusts that are representative for the last 3 h.

[23] A crucial factor for simulating the NLLJ is the treatment of the BL. The turbulent transport in stable BLs in ERA-Interim is parametrized by a K-diffusion scheme [*Beljaars and Viterbo*, 1999]. Artificially higher values for the diffusion coefficient K are used for stable BLs in order to achieve a better overall performance of the numerical weather prediction system [*Bechtold et al*., 2008; *Sandu et al*., 2013]. Undesired side effects are too smooth vertical profiles of meteorological variables in the nocturnal BL and comparably weak and higher residing NLLJs [*Sandu et al.*, 2013]. Further improvements in the representation of stable BLs in the ECMWF model is subject of ongoing research.

[24] The increased turbulent mixing within inversion layers [*Bechtold et al.*, 2008] affects the time and amount of momentum transfer to the surface. The associated momentum loss at the NLLJ level decelerates or even erodes the jet in the course of the night. The NLLJ strength in ERA-Interim during the morning is, therefore, likely to be underestimated. This can have an impact on the simulated diurnal cycle of the near-surface wind speeds and the mineral dust emission. Nevertheless, reanalysis give the best estimate of the past state of the atmosphere. Observation records with comparable resolution and record length are not available for this part of the world.

### 3.2 Observations

[25] The horizontal wind speed in the lowest 1500 m agl in ERA-Interim and observations are compared at different locations across North Africa to evaluate the representation of NLLJs. Radiosondes launched during the African Monsoon Multidisciplinary Analysis (AMMA) [*Redelsperger et al*., 2006; *Parker et al*., 2008] in 2006 and quality controlled by *Nuret et al*. [2008] are used here. The sites Agadez in Niger (16°N,7°E), Tombouctou in Mali (16°N,3°W), and Niamey in Niger (13°N,2°E) have been chosen for the validation. These locations have sufficiently homogeneous surroundings, frequent launches during the night, and lie within NLLJ hot spots (section 5). Radiosondes that were launched close to the three-hourly model data are selected, i.e., for midnight soundings 22.30 UTC to 01.30 UTC. Each of the preselected profiles is manually examined for the occurrence of a NLLJ. If a NLLJ is found, then it is matched with the corresponding profile from ERA-Interim in the grid box enclosing the station. In addition to AMMA radiosondes, the vertical wind profile at Chicha in Chad (16.9°N, 18.5°E) is analyzed for March 2005, when wind speed observations with pilot balloon (PIBAL) tracks from the Bodélé Dust Experiment (BoDEx) [*Todd et al.*, 2008; *Washington et al*., 2006] are available.

### 3.3 Detection of NLLJs

[26] NLLJs are detected in the ECMWF ERA-Interim reanalysis and forecasts at all available times by using a newly developed automatic detection algorithm. Two desirable key characteristics of this algorithm are the following: (1) the terrain independent identification that enables determining the exact wind speed and height of the NLLJ core and (2) the consideration of the reduced frictional effects for the nocturnal acceleration in upper levels. These features have not been combined in a single detection method for LLJs in previous studies (section 2.2). The first characteristic is implemented by choosing data on the original model levels from ERA-Interim. Depending on the surface pressure, the depth of the three lowest model layers is 18–22 m, 27–33 m, and 39–48 m, which is assumed to provide a sufficient vertical resolution of the BL. Using meteorological fields on model levels has the additional advantage of avoiding interpolation uncertainty. The second characteristic is achieved by requiring the presence of a surface inversion.

**Figure 2:**
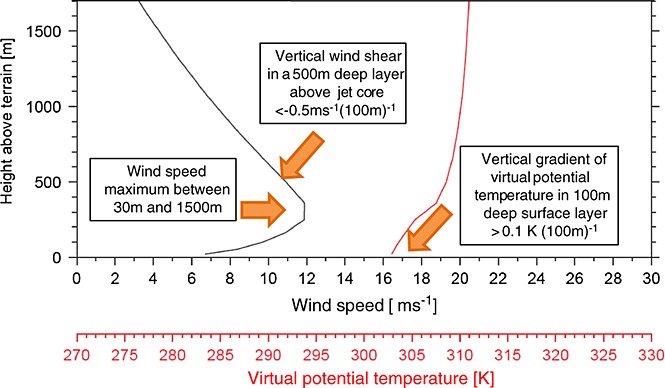
Schematic diagram showing the criteria for the NLLJ detection with an example of the vertical profiles of wind speed and virtual potential temperature from six-hourly ERA-Interim reanalysis.

[27] The main criteria of the detection algorithm in the present study are summarized in Figure 2. A NLLJ is identified as a wind speed maximum between the lowest model level and approximately 1500 m agl (1) that is situated above a stably stratified surface layer of at least 100 m depth measured by a vertical gradient of the virtual potential temperature exceeding 0.001 K m^−1^and (2) has a vertical wind shear stronger than − 0.005 s^−1^in a 500 m deep layer above the jet core.

[28] The first criterion reflects the reduced frictional effects in the nocturnal BL as a necessary prerequisite for the formation of a NLLJ (section 2.1). The choice of this rather weak stratification threshold enables the detection of cases with turbulent mixing beneath the NLLJ (section 2.3). Using a criterion for the vertical wind shear above the jet core confines wind maxima to vertically narrow jets which is suggested by *Stensrud* [1996]. This wind shear criterion implies a wind speed of at least 2.5 ms^−1^in the jet core. The height range of up to 1500 m is a generous definition of possible NLLJ heights (section 6.1) that is in agreement with the identification by *Lothon et al*. [2008]. The NLLJ height corresponds to the upper boundary of the model grid box where the maximum wind speed is found and is calculated using the time-dependent surface pressure.

[29] A night is defined as a “NLLJ night” if a NLLJ occurred between 18 and 06 UTC in the reanalysis. In order to analyze the evolution of NLLJs and the contribution to mineral dust emission after sunrise, NLLJs and additionally “NLLJ survivors” are detected in ECMWF forecasts. “NLLJ survivors” are defined as wind speed maxima which fulfill the criterion for the vertical wind shear 3 h after the occurrence of a NLLJ or a previous NLLJ survivor.

[30] The choice of the threshold values in the NLLJ detection algorithm is subjectively derived from ERA-Interim reanalysis. Possible uncertainties are assessed by sensitivity tests. The main findings are the following:

1. [1] An increased threshold for the vertical gradient of the virtual potential temperature from 0.001 K m^−1^to 0.01 K m^−1^reduces the number of identified NLLJs by about 60% in the annual and spatial mean. Dropping the criteria of the virtual potential temperature gradient leads to a spatially and annually small mean increase of 7% of the NLLJ frequency at 00 UTC, but this increase is inhomogeneously distributed across the continent. In this test, NLLJs at 00 UTC in regions of baroclinic conditions are pronounced, e.g., along the West African monsoon front and along coasts. The increase of detected LLJs is larger during the day when surface inversions are usually absent. Since reduced frictional effects at night are expected to enable a nocturnal acceleration of a LLJ (section 2.1), but cases with turbulence beneath the NLLJ need to be included to investigate dust emission associated with NLLJs, the weak stability criterion of 0.001 K m^−1^is chosen.
2. [2] Reducing the mean vertical wind shear to −0.0025 s^−1^in the 500 m deep layer increases the number of NLLJs across the domain by up to 80%. In contrast, requiring −0.005 s^−1^in a shallower layer of 300 m decreases the number of jets by up to 50%. Since the geographical structures of the NLLJ occurrence frequency is robust, the intermediate threshold seems to be a reasonable approach that successfully excludes NLLJs of a large vertical extent.

[33] The result showing an almost unchanged areal extent of the NLLJ hot spots but fairly large frequency changes with different thresholds for the vertical wind shear is similar to findings from LLJ detections in other studies. For instance, *Bonner* [1968] shows that the regions of most frequent LLJ occurrence over the United States remains similar, but the actual number of identified jets varies by a factor of six when the threshold values for identifying NLLJs are changed. These tests clearly underline the sensitivity of the method to the choice of threshold values.

### 3.4 Offline Dust Model

[34] Since ERA-Interim does not have a prognostic aerosol scheme, mineral dust emission is calculated offline with the mineral dust emission model developed by *Tegen et al*. [2002]. The model is driven by 10 m wind speeds and the moisture content in the uppermost soil layer from ECMWF ERA-Interim forecasts. Soil particles are mobilized in a source, when the near-surface wind speed exceeds the particle size-dependent threshold velocities [*Marticorena and Bergametti*, 1995]. The vertical dust emission flux is calculated from the horizontal particle flux, which is a function of the friction velocity cubed [see further *Tegen et al*., 2002].

[35] Potential sources for mineral dust aerosol are prescribed by the dust-source-activation frequency map by *Schepanski et al.* [2009], where sources are identified from the MSG SEVIRI infrared dust index product [*Schepanski et al.*, 2007]. A grid box of 1°×1° is defined as a potential dust source if at least two dust events were observed between March 2006 and February 2008. The surface roughness length of dust sources is set to a constant value of 10^−3^ cm as in *Schepanski et al.* [2007]. The activation of dust sources is limited to nonsaturated soils and expressed by soil moisture values below the field capacity. Dust sources, i.e., silt and clay soil types, are assumed to have a field capacity of 0.28 m^3^ m^−3^. The sensitivity to this parameter has been tested and was found to be negligible in the Sahara, which is in agreement with previous literature [*Laurent et al.*, 2008]. Dust emission is parametrized for four soil particle-size distributions, namely coarse sand (500–1000 μm), fine and medium sand (50–500 μm), silt (2–50 μm), and clay (0–2 μm). The relative content of the different populations has been derived from the global soil-texture data from the Food and Agriculture Organization on a horizontal grid of 0.5°. The threshold 10 m wind speed is determined for each of the four particle size bins. This threshold 10 m wind speed for dust emission is not parameterized as a function of the NLLJ wind speed.

## 4 Validation

### 4.1 Annual Cycle of NLLJ Characteristics

**Figure 3:**
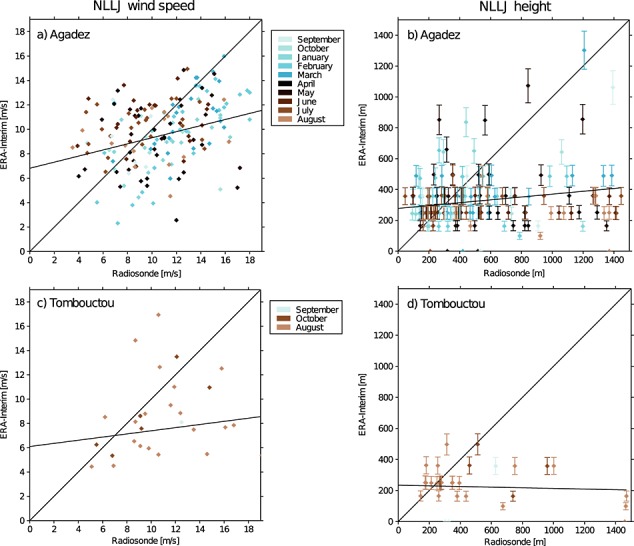
Scatter plots for validation of NLLJs in ERA-Interim forecasts at 00 UTC in different months of 2006. Column on the left shows the NLLJ wind speed for (a) Agadez and (c) Tombouctou, and on the right the NLLJ height with error bars indicating the calculated model layer thickness for (b) Agadez and (d) Tombouctou, based on radiosondes launched during AMMA and ERA-Interim forecasts initialized at 12 UTC on the previous day.

[36] The representation of NLLJ characteristics in ERA-Interim is tested with quality-controlled radiosondes launched during AMMA at 00 UTC at Tombouctou for August–October 2006 and at Agadez for January–October 2006. Figures a–b show the wind speed and the height in the jet core from ERA-Interim forecasts against radiosonde ascents at Agadez. The wind speed in the NLLJ core in Figure 3a shows a large spread. Core wind speeds larger than 9 m s^−1^are underestimated by the model. NLLJ wind speeds between September and March are too small, while the results for the remaining year are less coherent. The former time period coincides with the occurrence of a NLLJ hot spot around Agadez (section 5), indicating that the strongest and highest NLLJs in this hot spot may be underrepresented in the statistics based on ERA-Interim. The scatter diagram for the height of NLLJs (Figure 3b) shows a similarly large spread with a tendency toward underestimation of the NLLJ height by ERA-Interim when the observed height exceeds 300 m agl. The performance for the NLLJ height does not change in the course of the year.

[37] Figures c–3d show scatter diagrams for the wind speed and height of NLLJ cores at Tombouctou for August–October 2006, the first 2 months of which coincide with a NLLJ hot spot over the region (section 5). Both the wind speed and the height of the jet core tend to be underestimated by the model for values larger than 7 m s^−1^(Figure 3c) and 200 m agl (Figure 3d), respectively, in agreement with the findings for Agadez. The spread of data points and the slope of the regression line is smaller at Tombouctou, but this can be an artifact of the smaller number of midnight radiosondes.

### 4.2 Nocturnal Evolution of NLLJ Characteristics

**Figure 4:**
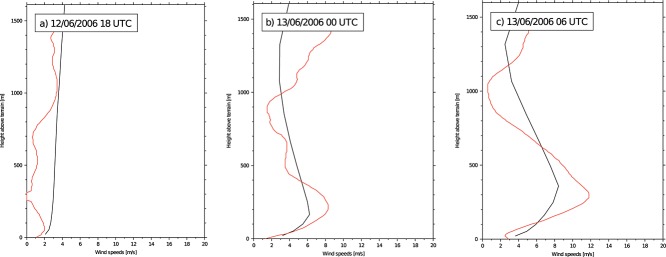
Vertical profile of horizontal wind speed at Niamey for (a) 12 June 2006 at 18 UTC, (b) 13 June 2006 at 00 UTC, and (c) 13 June 2006 at 06 UTC based on radiosondes (red) and ERA-Interim forecasts (black).

[38] Temporally higher resolved data is available at Agadez and Niamey in June 2006. These stations are located along the southern boundary of the Saharan heat low, where NLLJs are frequently embedded in the southerly inflow with the West African monsoon (section 5). Figure 4 shows an example for the development of a NLLJ at Niamey for the night of the 12–13 June 2006. While the observations show a jet of 11 m s^−1^, the model has a core wind speed of only 8 m s^−1^at 06 UTC.

**Figure 5:**
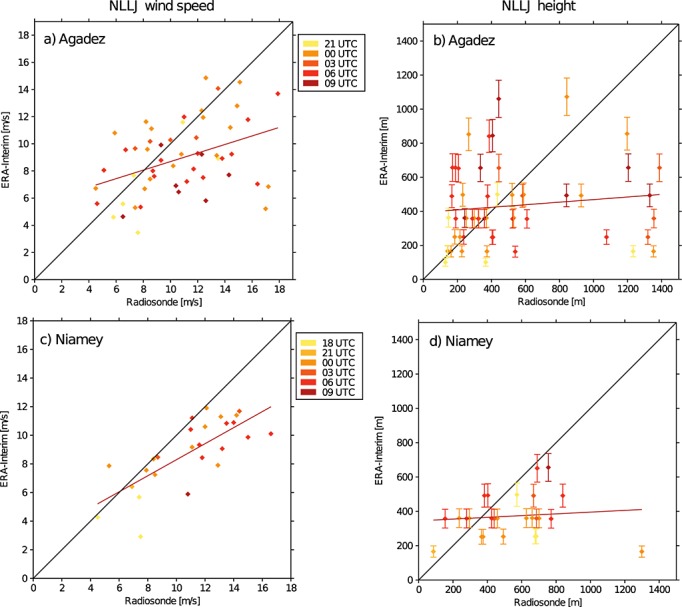
Scatter plots for validation of NLLJs in ERA-Interim forecasts for different times of the night in June 2006. Column on the left shows the NLLJ wind speed for (a) Agadez and (c) Niamey, and on the right the NLLJ height with error bars indicating the calculated model layer thickness for (b) Agadez and (d) Niamey, based on radiosondes launched during AMMA and ERA-Interim forecasts.

[39] Differences between model and observation for the height and speed of the NLLJ core throughout the night are seen in most profiles at Agadez and Niamey (Figure 5). The data at Niamey suggests that the height and speed of NLLJ tends to be underestimated when they reside above 400 m agl and when they are faster than 6 m s^−1^(Figures 3c–3d). Although the sampling size is not ideal for drawing a clear conclusion, the data suggests that the underestimation of wind speed in the jet core is growing over night. This would imply that the necessary acceleration of the jet in the course of the night is not well captured by ERA-Interim. However, the underestimation of the height of the NLLJ does not show a comparable temporal dependency, and there is no clear diurnal dependency at Agadez (Figures 3a–3b). The data at Agadez and Niamey show that the fast and high NLLJs are underestimated in ERA-Interim in line with the results for the annual cycle of the NLLJ characteristics.

### 4.3 Dust-Emitting NLLJs

[40] The Bodélé Depression is a hot spot for the occurrence of NLLJs and dust emission. This region holds fine sediments that are prone to wind erosion making it the most active source for dust aerosol worldwide [*Todd et al*., 2008, and references therein]. The BoDEx field campaign (BoDEx) [*Todd et al*., 2008; *Washington et al*., 2006] monitored the conditions of this remote place by pilot balloon (PIBAL) measurements at Chicha for the period of 28 February to 13 March 2005. Although the applied theodolite technique for tracking the balloon has larger uncertainties than a radiosonde measurement, the unique data from BoDEx is an independent benchmark for evaluating the NLLJ and dust emission.

#### 4.3.1. NLLJ

**Figure 6:**
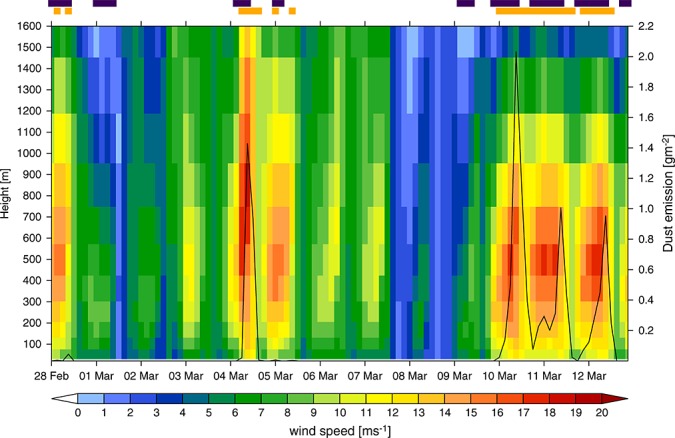
Time series for the low-level profile of horizontal wind speed at Chicha for 28 February to 13 March 2005. Horizontal wind speed (shaded) and time of automatic NLLJ detection (black bar) from ERA-Interim forecasts. Dust emission flux (contour) and time of DSA (orange bar) based on the dust model by *Tegen et al.* [2002] and ERA-Interim forecasts.

[41] Figure 6 shows the vertical profile of horizontal wind speed in ERA-Interim during the BoDEx time period. This time series from the model clearly indicates the presence of a NLLJ in all nights, although the NLLJs for 1–2 March 2005 and 8–9 March 2005 are rather weak with less than 10 m s^−1^. The development of the strongest NLLJ in this time period was forecasted for 4 March 2005 with a core wind speed of 19 m s^−1^. The same day provides a good example for the downward mixing of momentum from the jet during the morning. The associated increase in near-surface wind speed from 6 m s^−1^ at night to 12 m s^−1^leading to dust emission during the morning of 4 March 2005 is apparent.

[42] The vertical structure of wind speed from ERA-Interim is remarkably similar to the profile observed with PIBAL shown by *Washington et al.* [2006] and *Todd et al*. [2008]. *Todd et al*. [2008] report a mean maximum wind speed of 17 ms^−1^with a mean jet core height at roughly 400 m agl. The typical height of NLLJs is well reproduced by ERA-Interim. The NLLJ core wind speed in ERA-Interim is, however, systematically lower, namely 24–50%. An underestimation of the wind speed in the NLLJ of 15% at 06 UTC is also found for a regional model that has been tuned for the regional characteristics by *Todd et al.* [2008]. This larger underestimation by the global ERA-Interim forecast system relative to observations than the regional MM5 might be explained by different factors including the coarser resolution of ERA-Interim, differences in the physical parameterization, and the lack of tuning of ERA-Interim to match the regional condition of the Bodélé Depression.

#### 4.3.2. Dust Emission

[43] The offline dust emission model by *Tegen et al.* [2002] driven with ERA-Interim wind speeds simulates dust emission on about half of the days during the BoDEx observation period. Days with and without dust emission are reproduced well. These dust emissions occur mostly during the mid-morning; the time of day when the downward mixing of NLLJ momentum to the surface is observed and simulated (Figure 6). During the first half of the BoDEx period, 0.05 g m^−2^dust is emitted in the morning of 28 February 2005 and 1.4 g m^−2^on 4 March 2005 based on the offline dust emission calculations. These dust emissions occur simultaneously with a NLLJ or NLLJ survivor that are detected by the automatic algorithm. The dust emission calculations further agree with the dust-free periods for 1–3 March 2005 and 6–9 March 2005 that have been observed at Chicha, when the large-scale horizontal pressure gradient over the region was weak [*Todd et al.*, 2008].

[44] On 10 March 2005, the large-scale meteorological conditions change with the rapid formation of a ridge over North Africa [*Todd et al.*, 2008]. ERA-Interim forecasts the associated NLLJs that are successfully identified by the automatic detection algorithm for 10–12 March 2005. PIBAL observation does not document these jets due to strong dust storms that prevented any measurements in that period [*Todd et al.*, 2008]. These dust storms are reproduced by the dust emission model with a peak emission of 2.1 g m^−2^during the mid-morning of 10 March 2005.

[45] In summary, the frequency of dust source activation, the general intensity classification of emission events, the frequency of NLLJ occurrence, and the NLLJ height observed during BoDEx [*Todd et al.*, 2008] are well simulated by the ERA-Interim forecast system and the offline dust emission model by *Tegen et al.*[2002]. The NLLJ height and wind speed tend to be underestimated by the model compared to the AMMA stations when the NLLJ resides higher than 200–400 m agl and is faster than 6–9 m s^−1^. This finding is in agreement with the evaluation of the nocturnal BL in ERA-Interim available from the literature (section 3.1).

## 5 NLLJ Climatology

**Figure 7:**
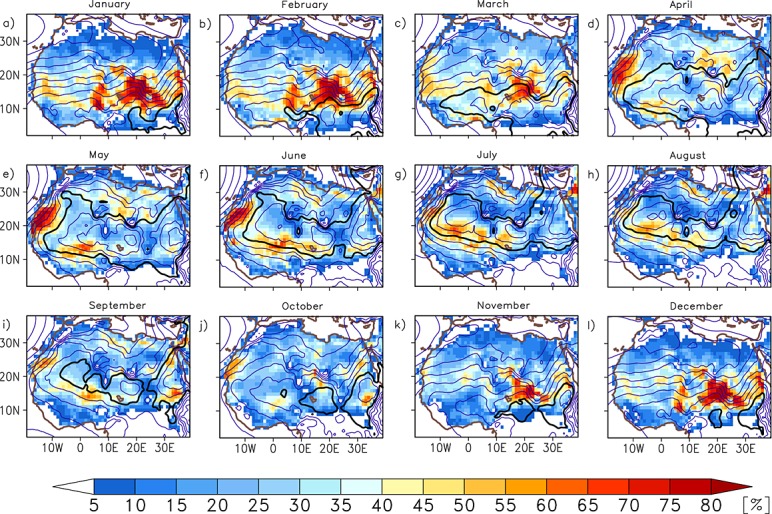
Annual cycle of the frequency of NLLJ nights. Monthly mean occurrence of nights with a NLLJ (colors) and mean 975 hPa geopotential height at 00 UTC (contours) for (a) January, (b) February, (c) March, (d) April, (e) May, (f) June, (g) July, (h) August, (i) September, (j) October, (k) November, and (l) December, based on six-hourly ECMWF ERA-Interim reanalysis 1979–2010 and the new NLLJ detection algorithm. Geopotential heights are contoured in steps of 1 gpdm (thick contours correspond to 30gpdm). Note that the 975 hPa level is below the model orography over parts of North Africa.

**Figure 8:**
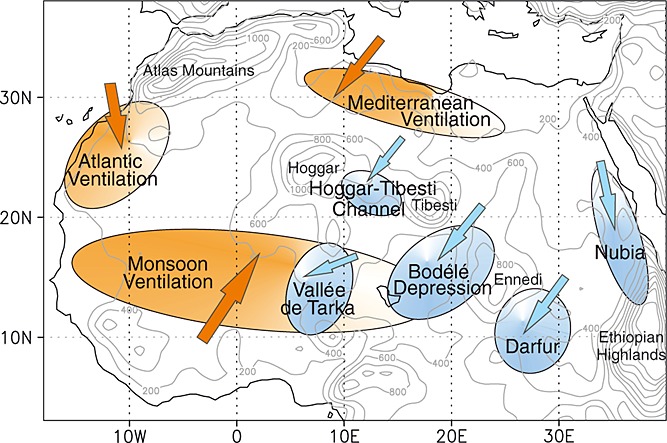
Overview of NLLJ hot spots in North Africa for November–February (blue) and April–September (orange). Contours show the terrain height in steps of 200 m. The arrows indicate the prevailing wind direction for each hot spot.

[46] NLLJs are a frequent phenomenon in North Africa. In the annual and spatial mean, NLLJs are detected in 29 +/− 4 % (mean +/− standard deviation) of the nights in the ERA-Interim reanalysis for 1979–2010 over North Africa. Figure 7 shows the seasonal cycle by the monthly mean frequency of NLLJ nights and the mean geopotential height at 975 hPa that is a strong control of wind speed at any fixed point with constant roughness. In January, NLLJs occur in 5–25% of all nights between 20°N and 30°N (Figure 7a). South of 20°N, the NLLJ frequency reaches higher values, typically 25% to 50%. The most active regions show NLLJ frequencies of up to 80%, namely the Bodélé Depression, the Vallée de Tarka, the Nubian desert, the Darfur region, and the Hoggar-Tibesti channel. These hot spots in terms of occurrence frequency, summarized as blue areas in Figure 8, remain active in February (Figure 7b) but decrease in their dominance in March (Figure 7c) before they disappear between April and September (Figures 7d–7i). In the latter period, the NLLJ climatology shows hot spots in the western Sahel and in areas along the Atlantic and the Mediterranean coast lines. Maxima during this time of the year are summarized as orange areas in Figure 8. NLLJs in the western Sahel and Sudan occur in 40–65% of the nights between April and September (Figures 7d–7i). The overall maximum for April–September is found in the Atlantic ventilation hot spot, which is named after the advection of relatively cool maritime air to hot areas further inland. Here, the amount of NLLJ nights is comparable to the frequency in the Bodélé Depression for November–March (Figures 7a–7c and 7k–7l). In contrast, NLLJs in the Mediterranean ventilation occur only during up to 60% of the nights.

**Figure 9:**
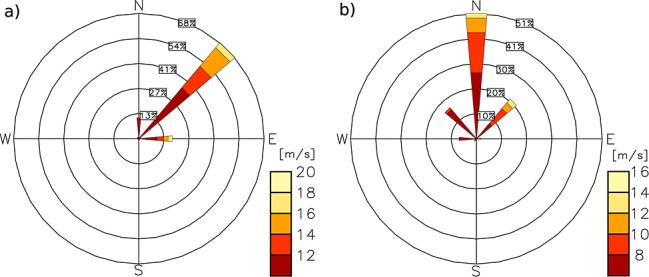
Wind roses for NLLJs (a) in the Bodélé Depression hot spot for November–March and (b) in the Atlantic ventilation hot spot for April–June, based on six-hourly ECMWF ERA-Interim reanalysis 1979–2010. Regions are defined in Figure 10a.

[47] Specific environmental conditions, such as (1) orographic channelling and (2) low-level baroclinicity, may favor the development of NLLJs (section 2.1). Previous studies suggest orographic channeling as driving mechanism for NLLJs over the Bodélé Depression [e.g., *Washington and Todd*, 2005; *Todd et al.*, 2008]. A climatology of the wind direction and speed in the core of NLLJs is depicted as a wind rose for the Bodélé Depression in Figure 9a. Here, the majority (68%) of the NLLJs are north-easterly between November and March. The narrow distribution around the prevailing wind direction indicates channeling of the north-easterly Harmattan winds between the Tibesti and Ennedi Mountains. This can be further supported by the horizontal gradient of the 975 hPa isohypses (Figures 7a–7c and 7k–7l), which show a ridge upstream of the channel and a trough in the lee of the Mountains. The air is accelerated downgradient and frequently forms NLLJs above the stably stratified surface layer at night. Half of the NLLJs in the Bodélé Depression hot spot are characterized by wind speeds of 12–20 m s^−1^. These core wind speeds agree well with the range of measured and simulated NLLJs in the area from *Todd et al.* [2008]. In addition to the Bodélé Depression hot spot, orographic channeling of the Harmattan winds might also play a role between the Hoggar and the Tibesti Mountains.

[48] Another NLLJ forming in response to the effects of orography is the jet along the northern slopes of the Ethiopian Highlands described by *Rife et al.* [2010] and part of the Nubian desert hot spot in Figure 8. The spatial distribution of the NLLJ index by *Rife et al.* [2010] is not directly comparable to the NLLJ frequency presented here, but the results by *Rife et al.* [2010] enable calculating a frequency of nonzero NLLJ indices of 94% in the Ethiopian NLLJ at 12.8°N and 34°E for January. The largest indices for the Ethiopian NLLJ by *Rife et al.* [2010] occur in the area 10°N–15°N and 30°E–38°E. Based on this spatial extend, the Ethiopian jet occurs in up to 70% of the nights in the climatology for January presented here. The difference between the results is caused by the different methods for the NLLJ identification (section 2.2).

[49] Between April and September (Figures 7d–7i), the location of NLLJ hot spots along the margins of the Saharan heat low point at favorable conditions due to low-level baroclinicity. NLLJs are, here, embedded in the large-scale inflow from the Mediterranean, the Atlantic, and in the West African Monsoon flow over the western Sahel. The Atlantic ventilation hot spot coincides with an increased horizontal gradient of the 975 hPa isohypses (Figures 7d–7i) between the Azores High and the northwestward expanding heat low. NLLJs are embedded in this inflow, most frequently between April and June (Figures 7d–7f). Deflection of the air masses by the Atlas Mountains causes prevailing northerly NLLJs (Figure 9b). Half of the NLLJs have maximum core wind speeds of 8–16 m s^−1^. The NLLJs between 15°N and 20°N have previously been described as part of the Atlantic inflow by *Grams et al.* [2010]. The results of the present study indicate that the Atlantic ventilation extends even further north to the southern foothills of the Atlas Mountains. Similarly, low-level baroclinicity may cause the Mediterranean ventilation hot spot over northern Libya between May and September (Figures 7e–7i). Here, the horizontal pressure gradient evolves between the ridge over the Mediterranean Sea and the Saharan heat low. NLLJs over the western Sahel follow the latitudinal migration of the heat low along with the West African Monsoon. This result shows that the frequent formation of NLLJs along the margins of the Saharan heat low, previously proposed by observational studies for parts of West Africa [*Parker et al.*, 2005; *Pospichal et al.*^2010^; *Abdou et al.*, 2010; *Bain et al.*, 2010], is not limited to the southern margins of the heat low.

## 6 Characteristics of NLLJs

### 6.1 Height and Core Speed

**Figure 10:**
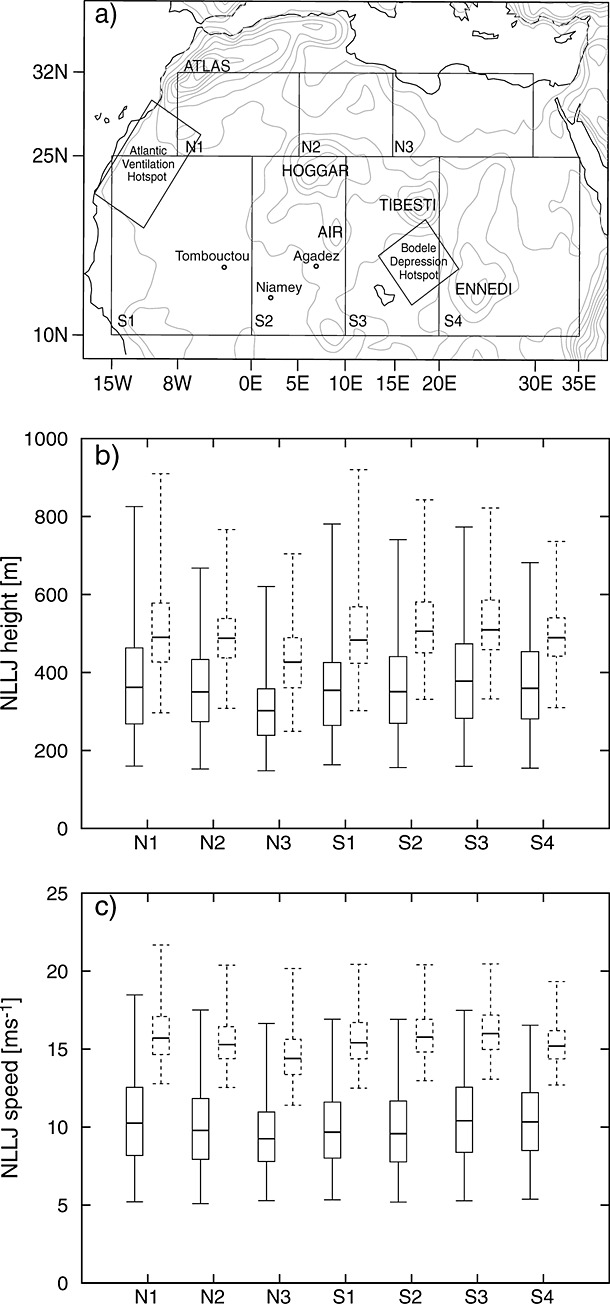
Climatology of NLLJ characteristics. (a) Geographical location of the subdomains (black boxes) and ERA-Interim model orography (grey) in 200 m steps. (b) Box-and-whisker plots for the core height and (c) core wind speed for all NLLJs (solid) and NLLJs emitting dust at the same time (dashed). Based on six-hourly ECWMF ERA-Interim reanalysis for 1979–2010.

[50] The detection algorithm enables a statistical assessment of the height and wind speed of NLLJs over North Africa. The median NLLJ height and core speed across North Africa is 350 m agl and 10 m s^−1^in the annual statistics. In order to identify regional differences, seven subdomains are defined (Figure 10a): three regions across the northern Sahara (N1, N2, and N3) and four subdomains across the southern Sahara and Sahel (S1–S4). The geographical placement of the subdomains is motivated by the sampling areas used by *Schepanski et al.* [2009]. The lowest NLLJs are found in the Mediterranean ventilation regions N3 with a median of 300 m agl and a 99% percentile of 620 m agl, followed by N2 with 350 m agl as median height and a 99% percentile of 670 m agl (Figure 10b). NLLJs over the Bodélé Depression, as part of S3, frequently reside at heights around 380 m agl (median) to up to 770 m agl (99% percentile). The regional differences for the NLLJ core wind speeds are shown in Figure 10c. NLLJs are fastest in N1 and S3 with up to 18 m s^−1^in the spatially averaged 99% percentile, but the overall difference between the subdomains is small.

[51] The statistics of NLLJs in North Africa represent observed conditions reasonably well. Validating ERA-Interim against PIBAL observation at Chicha indicates that the statistic for the height of the NLLJ in S3 is well represented (section 4). The core speed, however, is underestimated by 37% averaged over the BoDEx period at Chicha. *Todd et al.* [2008] uses another reanalysis data set to determine a maximum core wind speeds of the NLLJ over the Bodélé Depression of 12 m s^−1^at 17°N, 19°E, which is close to the upper quartile of the S3 subdomain. *Rife et al.* [2010] finds core speeds of 12 m s^−1^and a height of 400 m agl for the Ethiopian NLLJ, which is close to the upper quartile of the statistics for S4 of the present work. Upper air measurements at Khartoum by *Farquharson*[1939] show a NLLJ in a mean level of 305 m agl and an annual mean wind speed of 9 m s^−1^. This observation lies close to the lower quartile of the computed NLLJ height and wind speed statistics of subdomain S4 which covers but is not limited to the Sudan. Heights and wind speeds from AMMA radiosondes (section 4) are well captured in S2 by ERA-Interim, but the high and fast jets are underrepresented in the statistics (section 4). The typical heights of the NLLJ of 200–400 m from observations at Niamey [*Abdou et al*., 2010; *Lothon et al*., 2008] is well in agreement with the presented statistic for S2. The wind speeds are in the same range but tend to be underestimated in S2 compared to wind profiler observations at Niamey by *Lothon et al.*[2008].

**Figure 11:**
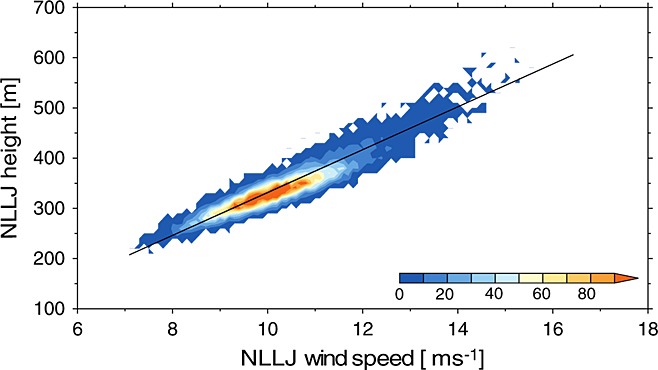
Scatter plot for NLLJ core heights against wind speeds at 00 UTC for North Africa, based on six-hourly ERA-Interim reanalysis for 1979–2010. Colors indicate the number of data pairs in the bin. Linear regression (solid line) is given by *f*(*x*)=−95.1+42.7*x* with a Pearson correlation coefficient R^2^=0.98.

[52] Core height and wind speed of NLLJs show a linear relationship (Figure 11). This finding is robust and not sensitive to single subdomains or seasons. At the same geographical latitude and for the same geostrophic wind speed, NLLJs can be expected to develop faster core wind speeds over longer roughness lengths. Variations of the background pressure gradient, the surface roughness, and the geographical latitude can, therefore, cause the spread around the linear regression. Figure 1 shows further that most NLLJs have intermediate heights of 300–350 m agl and wind speeds of 9–11 m s^−1^. Fewer jets are observed at the lower and upper end of the distribution. Assuming calm conditions at the ground and a constant stratification, a strong NLLJ closer to the surface is more likely to become dynamically unstable, so that the NLLJ momentum is transferred to lower levels by turbulence. Stronger NLLJs close to the surface are therefore less likely to be detected in the temporally coarse resolution data. The less frequent occurrence of strong NLLJs at higher altitudes can be linked to a dependency of the jet height on the depth of the surface inversion layer [*Blackadar*, 1957; *Baas et al.*, 2009] and the strength of the geostrophic wind [*Gross*, 2012]. The growth of the surface inversion can be disturbed by turbulent mixing in the course of the night, which will be further explored in the following section.

### 6.2 Nocturnal Evolution

**Figure 12:**
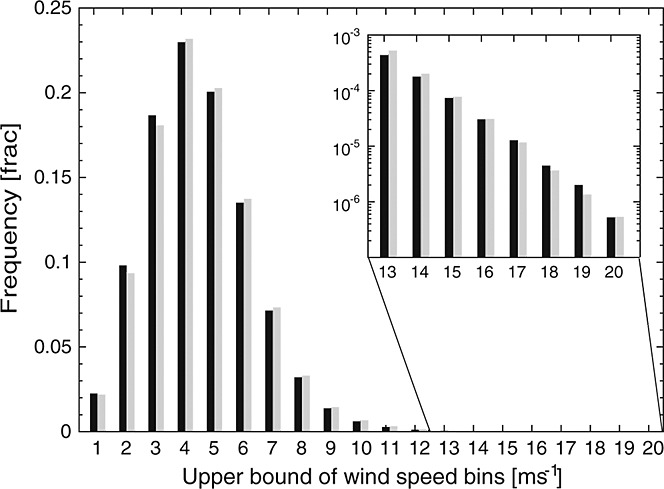
Frequency distribution of the 10 m wind speeds at 18 UTC, 00 UTC, and 06 UTC from ECMWF ERA-Interim six-hourly reanalysis (black) and three-hourly forecasts (grey) over North Africa for 1979–2010. Note the logarithmic scale in the zoom for the high end of the wind speed distribution, which is relevant for mineral dust emission.

[53] Shear-induced turbulence beneath a NLLJ affects BL characteristics with implications for the diurnal cycle of mineral dust emission. ECMWF ERA-Interim forecasts are used for investigating the nocturnal evolution of NLLJ characteristics with higher temporal resolution. The frequency distribution of the near-surface wind speed from the forecasts are compared to ERA-Interim reanalysis at 18 UTC, 00 UTC, and 06 UTC as spatial mean over North Africa in Figure 12. Differences at the high end of the frequency distribution are negligible, which encourages the usage of the temporally finer resolved ERA-Interim forecasts for NLLJ process studies.

[54] Figure 3a shows the nocturnal evolution of the fraction of grid boxes that have a NLLJ or a NLLJ survivor. At 18 UTC, NLLJs are found in 10% of the grid boxes over North Africa, which is defined as an area covering all seven subdomains plus the edges: 15°W–35°E and 10°N–32°N. Jets at this time of day are predominantly found in the east of the domain (not shown). As the night progresses, the number of grid boxes with NLLJs increases and reaches the maximum of 80% at 03 UTC. The subsequent decrease of the number of NLLJ grid boxes is linked to the onset of the NLLJ breakdown in the east, where 06 UTC is after local sunrise. NLLJs survive in 30% of all North African grid boxes at 09 UTC and 5% at 12 UTC; the latter of which are limited to the western boundaries of North Africa. The NLLJ statistic at 15 UTC is not shown due to the small sampling size at this time of day.

**Figure 13:**
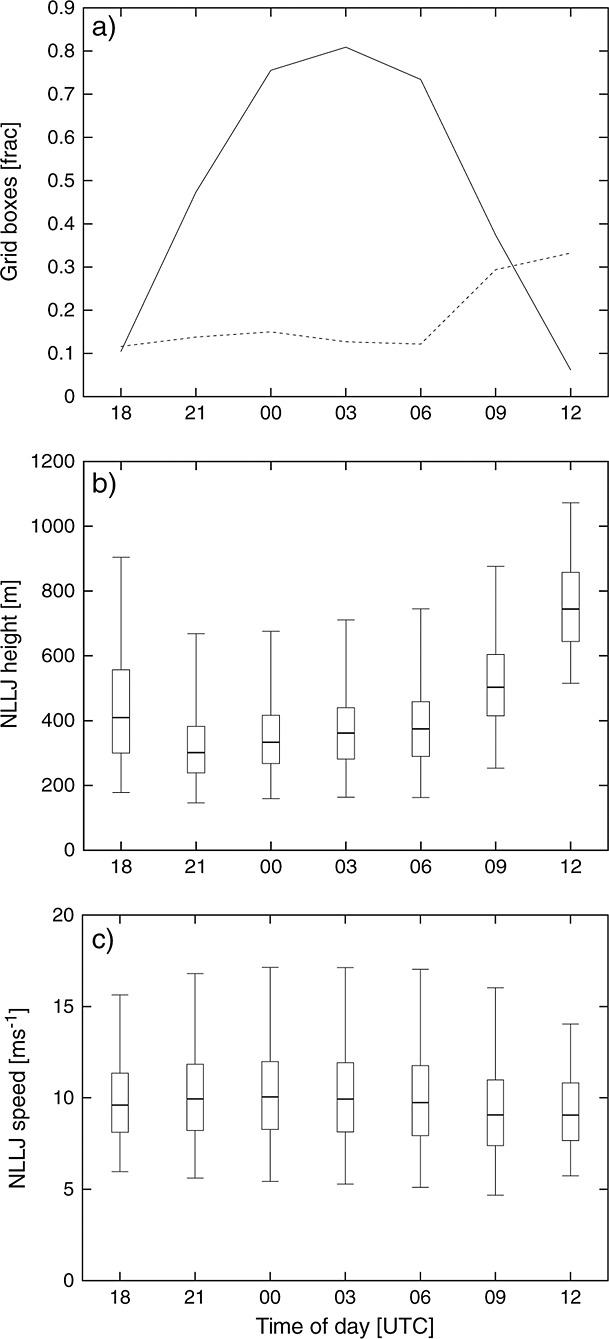
Temporal development of NLLJs and NLLJ survivors over North Africa. (a) Nocturnal cycles of the mean fraction of grid boxes with a NLLJ (solid) and DSA (dashed), box-and-whisker plots showing 99%, 75%, 50%, 25%, and 1% percentiles of (b) the NLLJ core height and (c) core wind speed as function of time in UTC. Based on three-hourly ECWMF ERA-Interim forecasts for 1979–2010.

[55] It is worth noting that the presented diurnal cycle is influenced by the difference between UTC and local time (LT). Local times across North Africa range from approximately UTC +2 in the east to UTC −1 in the west. In eastern regions, 09 UTC corresponds to 11 LT which is too late for the expected NLLJ breakdown at 09 LT. In fact, 11 LT in the west (12 UTC) clearly shows an abrupt reduction in the number of NLLJ survivors. Here, 09 UTC corresponds to 08 LT which can be too early for the NLLJ breakdown. This means that the peak in the near-surface wind speed is not captured in all areas. The associated mineral dust emission is, therefore, likely to be underestimated.

[56] The temporal development of the NLLJ core height and wind speed is shown in Figures 3b–3c. Between 18 UTC and 21 UTC, the median NLLJ height decreases from 400 m agl to 300 m agl, while the median core wind speed increases from 9 m s^−1^ to 10 m s^−1^. The wind speed increase indicates an acceleration of NLLJs. Decreasing spatial mean heights of the NLLJ are linked to generally shallow inversion layers at the beginning of the new NLLJ generation across large areas at 21 UTC. The NLLJ formation at 18 UTC is limited to S4 in all months and S3 between December and February, where the NLLJs reside relatively high (not shown).

[57] The nighttime development between 21 UTC and 06 UTC shows an increase of both the core height and the number of NLLJ grid boxes, which points to an ongoing NLLJ evolution. However, the NLLJ wind speed does not show the expected acceleration in the course of the night. This hints at downward mixing of momentum due to shear-induced turbulence during the night. If the static stability is not high enough for balancing the vertical wind shear, then turbulence transfers momentum to lower levels. The associated loss of momentum at and beneath the NLLJ nose decelerates the jet or even erodes the wind speed maximum. New NLLJs may start to form where they have been eroded earlier in the night. Both the weakening of pre-existing NLLJs and the beginning of new NLLJ formations explains the missing increase of the jet wind speed in the statistic. The decreasing number of grid boxes with a NLLJ after 03 UTC suggests that NLLJs are eroded and that less new NLLJs form toward the end of the night. The increasing height of NLLJs during the night can be linked to the depth of the surface inversion [*Blackadar*, 1957; *Baas et al.*, 2009]. The inversion depth depends on both the cooling rate, determined by the net radiation budget at the surface, and the entrainment of air from the residual layer, under constant environmental conditions. Entrainment can be efficient during shear-driven turbulence beneath a NLLJ near the top of the surface layer [*Van de Wiel et al.*, 2010]. It is this entrainment due to the vertical mixing beneath the NLLJ that can increase the inversion depth and therefore lift the NLLJ core to higher altitudes.

[58] The NLLJ core wind speeds decrease and the height increases after 06 UTC. In combination with a decreasing number of grid boxes with NLLJs or NLLJ survivors, this development illustrates the expected breakdown and erosion of NLLJs during the following mid-morning. Dust emission due to the downward mixing of NLLJ momentum occurs when the increase in the 10 m wind speed is sufficiently high to exceed the threshold for dust mobilization.

### 6.3 Characteristics During Emission

[59] The distributions of the NLLJ core wind speed and height are different during dust emission events as has been calculated by the offline dust emission model (Figures 10b–10c). Dependent on the subdomain, the median NLLJ speed and height during dust emission ranges from 14−16 m s^−1^, and 430–510 m agl, respectively. The distributions of the dust-emitting subsample of NLLJs are shifted to the upper quartile of the distributions of the enclosing NLLJ sample in all subdomains. In some regions, even the 25% percentile of wind speed of dust-emitting NLLJ is shifted to the upper quartile of the background climatology. This shift reflects the necessity of low-level wind speeds exceeding the threshold value for dust emission onset. The 1% percentile of core wind speed of dust-emitting NLLJs has values of 12–13 m s^−1^at relatively low levels of 250–350 m, which can be interpreted as the threshold NLLJ characteristics for mineral dust emission.

## 7 NLLJs-Generating Dust Emission

[60] The near-surface winds from the ECMWF ERA-Interim forecasts generate a total annual dust emission flux of 428 +/− 46 Tg for 1979–2010 over North Africa. This dust emission amount lies within the computed dust emission range of 130–1600 Tg a^−1^over North Africa from previous studies [*Engelstaedter et al.*, 2006, and references therein]. The most recent assessments of the mean annual dust emission in North Africa are the following: 2077 Tg a^−1^for 2006 estimated by *Schmechtig et al.* [2011], 670 +/− 60 Tg a^−1^for 1996–2001 by *Laurent et al.* [2008], and 400–2200 Tg a^−1^from the AeroCom model intercomparison for 2000 [*Huneeus et al.*, 2011]. Most dust aerosol from North Africa is emitted during northern hemisphere spring with 41%, followed by winter with 29%, summer with 20%, and fall with 10% of the annual emission flux. Spatially integrated dust emission fluxes over North Africa and different subdomains are shown in Table 1. The choice of the three subdomains is, here, motivated by dominant dust sources in terms of emitted mass.

**Table 1:** Estimate of Mean Mineral Dust Emission in Tg^a^

	Region				
Time of Year	Bodélé Depression	North Sahara	West Sahara	North Africa
December–February	24	57	10	124
March–May	14	104	13	174
June–August	2	26	39	87
September–November	8	18	8	42
Annual	48	205	70	428

a Based on ECMWF ERA-Interim forecasts and the offline dust model by *Tegen et al.* [2002] for 1979–2010.

### 7.1 Seasonal Climatology

[61] Seasonal and spatial variations of the mineral dust emission are shown in Figure 4. From December to February (Figure 4a), a maximum of dust emission is found over the Bodélé Depression with typical dust emission values around 50 g m^−2^. The seasonal mean over the area from 15°N–19°N and 15°E–20°E is 24 Tg (Table 1). The dust emission amount from this source decreases as the year progresses to 14 Tg for March–May down to a minimum of 2 Tg in June–August. Between September and November, the seasonal mean dust emission increases again to 8 Tg.

**Figure 14:**
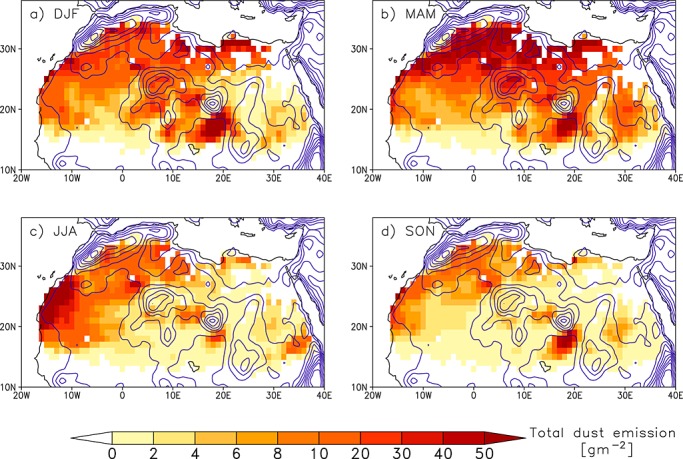
Dust emission climatology. Seasonal mean dust emission for (a) December–February, (b) March–May, (c) June–August, and (d) September–November, based on three-hourly ECMWF ERA-Interim forecasts for 1979–2010. Contours show the terrain height in steps of 200 m.

[62] Regions along the northern margins of the Sahara desert have the highest mineral dust emission of around 50 g m^−2^ between March and May compared to mostly 6–30 g m^−2^in the rest of the year (Figure 14b). In northern hemisphere spring, the dust emitted in the area from 25°N–35°N and 10°W–25°E contributes 104 Tg or 25% of the annual total dust emission budget of North Africa. The dominant dust sources between June and August are found in western North Africa with values between 20 g m^−2^ and 50 g m^−2^(Figure 14c). Here, 39 Tg of dust aerosol is emitted in the seasonal mean over the region covering 20°N–28°N and 18°W–10°W. The three dominant dust sources contribute 73–81% of the seasonally averaged dust emission over North Africa (Table 1). The relative importance of individual sources for dust emission varies seasonally.

**Figure 15:**
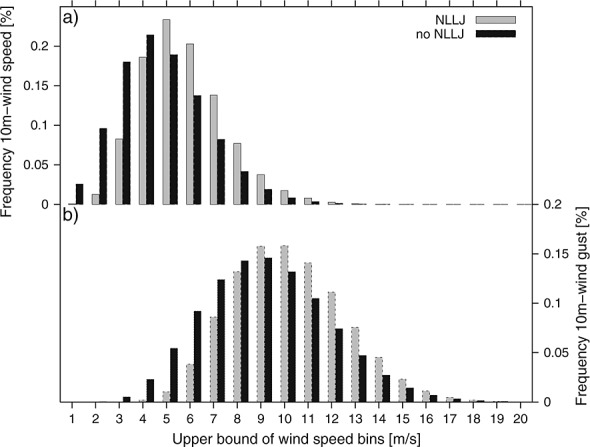
Frequency distribution of the 10 m wind speed during the mid-morning. Spatially averaged frequency distribution over North Africa of (a) the instantaneous 10 m wind speed at 06 UTC and 09 UTC and (b) the 10 m wind gusts at 09 UTC and 12 UTC when a NLLJ or a NLLJ survivor has been detected (grey), and when no NLLJ structure has been identified (black), based on three-hourly ECMWF ERA-Interim forecasts for 1979–2010.

[63] The new NLLJ detection algorithm in the present study enables for the first time a calculation of the relative contribution of NLLJs to the dust emission amount in a quantitative manner. Other dominant meteorological drivers for generating peak winds and, therefore, dust emission are assumed to be negligible during the occurrence of a NLLJ or a NLLJ survivor. The validity of this assumption is assessed by the statistical analysis of 10 m wind speeds at times when a NLLJ event is detected and when no NLLJ is present. Figure 15 shows the frequency distribution of both the instantaneous 10 m wind speed and the 10 m wind gusts during the mid-morning spatially averaged over North Africa. It is the mid-morning when the largest impact of NLLJ momentum on the near-surface wind speed and dust emission is expected (section 2.3). During the occurrence of NLLJs and NLLJ survivors, the 10 m wind speed and the gustiness shows a distinct shift toward higher wind speeds compared to the distribution when no NLLJ is simultaneously detected. This result from the frequency distribution can be interpreted as the linkage between the occurrence of a NLLJ and dust emission, since the latter is a function of the 10 m wind speed.

[64] It is interesting to note that the upward shift of the wind speed characteristics during the presence of a NLLJ or a NLLJ survivor during the mid-morning is largest in bins of medium wind speeds, 5–9 m s^−1^ for the 10 m wind speed and 9–14 m s^−1^for the gustiness, respectively (Figure 15). The tail of the frequency distributions shows only small differences between NLLJ and no NLLJ cases. This finding points toward a similar importance of NLLJs and other meteorological processes for the generation of the highest peak winds between 06 and 12 UTC. One of these processes can be a strong large-scale forcing that regularly transfers momentum to the surface. Even under these conditions, NLLJs can insert a diurnal variation of the near-surface wind speed but cause a smaller diurnal amplitude (section 2.1). The frequent vertical mixing under strong background flows with an associated disruption of the nocturnal LLJ enhancement and the occurrence of other meteorological processes generating peak winds explain the rather weak difference of NLLJs and other events for generating wind speeds at the upper end of the distribution.

**Figure 16:**
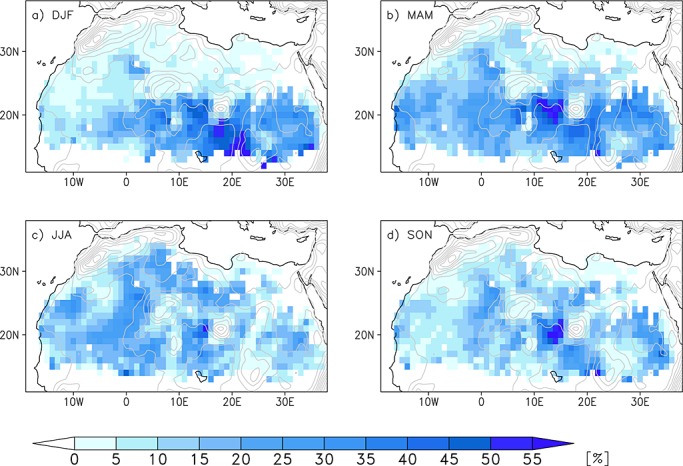
Seasonal mean NLLJ contribution to dust emission for (a) December–February, (b) March–May, (c) June–August, and (d) September–November, based on three-hourly ECMWF ERA-Interim forecasts for 1979–2010. Contours show the terrain height in steps of 200 m.

[65] The coherence of increased 10 m wind speeds during NLLJ events is used in the following to estimate the NLLJ contribution to dust emission which is based on the simultaneous occurrence of dust emission and a NLLJ or a NLLJ survivor. In the annual and spatial mean over North Africa, 15% of dust is emitted when NLLJ momentum mixing occurs. The seasonal mean climatology of the NLLJ contribution to dust emission in Figure 6 shows distinct regional characteristics. Between December and February, the NLLJ contribution is largest south of 20°N and east of 0° (Figure 16a). The expected peak of the NLLJ contribution to dust emission over the Bodélé Depression is well reproduced with up to 60% of the dust generated by NLLJs. This region remains distinctive in the climatology for March to May with 30–40% NLLJ contribution (Figure 16b), but the largest contribution of around 50% is now shifted to areas south of the Hoggar-Tibesti channel. At the same time, the relative importance of NLLJs over the western Sahel increases to values of 10–35%. From June to August, NLLJs contribute more substantially to dust emission north of 20°N with regional maxima of up to 35% over Algeria, Mali, Mauritania, and Libya (Figure 16c). Between September and November, the relative importance of NLLJs decreases in most regions in the north and west. In contrast, an increase of the NLLJ contribution to dust emission is found south of the Hoggar-Tibesti channel, over the Bodélé Depression, and over Nubia, where NLLJs contribute regionally 30–50% to the dust emission (Figure 16d).

### 7.2 Diurnal Cycle

[66] The diurnal cycle of the NLLJ contribution to dust emission is analyzed to understand the relative importance of NLLJ momentum for nighttime and morning dust emission. One perspective on the diurnal variations is given by the number of grid boxes with active dust emission in Figure 13a. The spatial mean number of dust-emitting grid boxes increases from 10% during the night to up to 30% in the mid-morning. The timing is consistent with the expected mechanisms for NLLJ momentum mixing, namely turbulence in the course of the night and the breakdown of NLLJs during the mid-morning. This suggests a mean relative importance of the NLLJ breakdown of up to 30% in terms of DSA frequencies without accounting for the dust emission amount. The diurnal cycle of DSA based on satellite observation, however, shows nighttime emission of 1–5% and morning DSA frequencies of 65% [*Schepanski et al.*, 2009]. This suggests an overestimation of nighttime and underestimation of morning DSA frequencies of the dust emission compared to the satellite observation, although the length of the time periods for the two climatologies differ. The relatively high nighttime DSA is in agreement with the too strong mixing in the BL at night (section 6.2).

**Figure 17:**
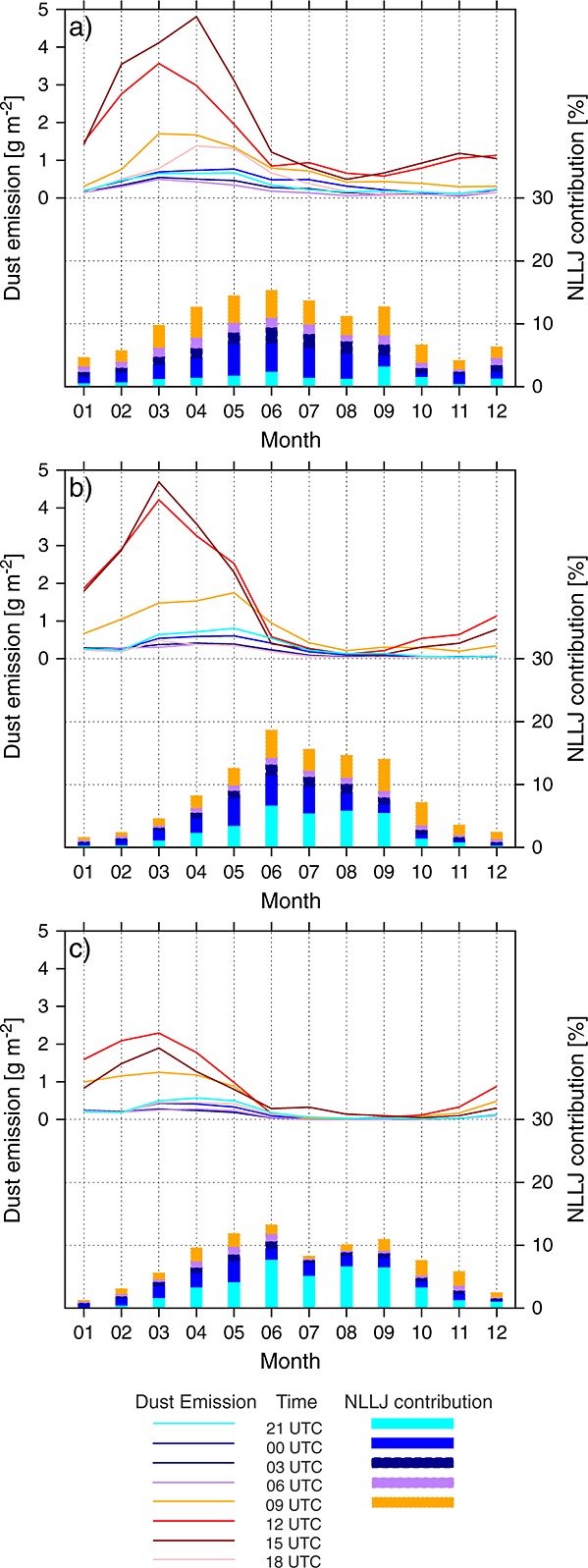
Contribution of NLLJs to mineral dust emission. Annual cycle of the monthly mean dust emission (lines) and the monthly mean of the relative contribution of NLLJs to the total dust emission (bars) at different times of the day (colors) as spatial mean per subdomains (a) N1, (b) N2, and (c) N3 based on three-hourly ECMWF ERA-Interim forecasts for 1979–2010. Regions are defined in Figure 10a.

**Figure 18:**
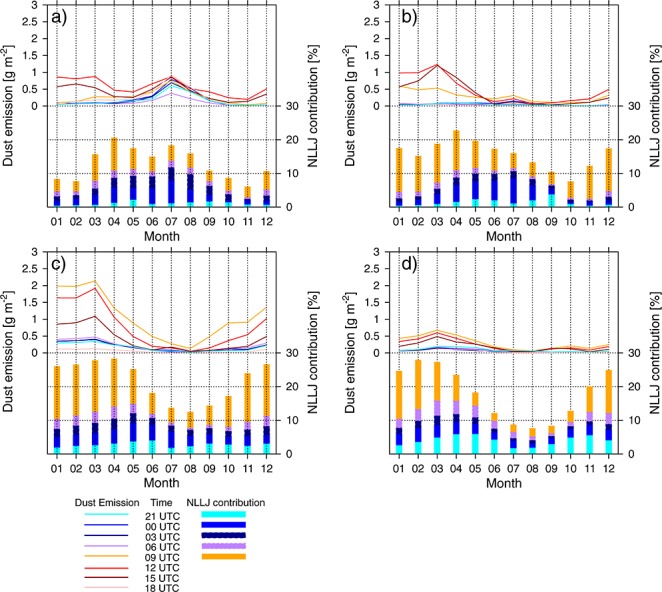
As Figure 7 for subdomains (a) S1, (b) S2, (c) S3, and (d) S4. Note the different scale for the dust emission.

[67] In order to analyze the annual and diurnal cycle of the NLLJ contribution in more detail, the approach based on the dust emission amount is used in the following. Figures 17 and 18 show the spatial mean annual cycle of the three-hourly dust emission at the top and the contribution of NLLJs to dust emission at the bottom for each of the seven subdomains given in Figure 10a. Whether NLLJs are a key driver for dust emission in specific regions can be concluded from considering (1) a phase comparison of the annual cycles of mineral dust emission and NLLJ contribution, (2) the total amount of dust emission, and (3) the frequency of NLLJs.

#### 7.2.1 Northern Subdomains

[68] In the northern subdomains, the spatial mean dust emission is largest in N1 (Figure 17a) and N2 (Figure 17b). The largest spatial mean dust emission is simulated during midday, up to 4 g m^−2^ at 12 UTC and 5 g m^−2^at 15 UTC, respectively. N3 has smaller midday maxima of dust emission with values around 2 g m^−2^for 12 UTC. Maxima at 09 UTC in March and May have values of 1–2 g m^−2^(Figures 17a–17c). Nighttime emission is substantially smaller with maximum values around 0.5 g m^−2^for 21 UTC and 00 UTC.

[69] NLLJ and NLLJ survivors generate 1–19% of the total dust emission flux in the spatial mean (Figures 17a–17c). Maximum contributions of 10–19% are found in N1 for March–September (Figure 17a), in N2 for May–September (Figure 17b), and N3 for April–June and August–September (Figure 17c). The overall maximum in the northern domains is 19% in N2 for June (Figure 17b). Here, NLLJ contributions remain high with values larger than 10% until September, which coincides well with the more frequent occurrence of NLLJs along the northern margins of the Saharan heat low in these months (section 5).

[70] N2 and N3 have larger values of the NLLJ contribution to dust emission at 21 UTC than at 09 UTC in most months, which points to turbulent mixing at night (Figures 17b–17c). The total dust emission fluxes at night are, however, small. Furthermore, the annual cycle of dust emission is not in phase with the NLLJ contribution cycle, and the large midday dust emission are not related to the breakdown of NLLJs. This indicates other processes as driving mechanisms along the northern margins of the Sahara. A potential underestimation of the NLLJ strength in the model [*Sandu et al.*, 2013], the temporal resolution, and the uncertainty in the NLLJ detection algorithm are likely to have a small impact on this result.

#### 7.2.2. Southern Subdomains

[71] Maxima of the spatial mean dust emission at single hours are generally smaller in the southern subdomains than the northern ones. For example, S1 and S2 emit 0.5–1 g m^−2^dust at 12 and 15 UTC between January and March. S1 has a secondary maximum in July, when the spatial mean dust emission reaches values of 0.4–1 g m^−2^at all times of the day (Figure 18a). Both western subdomains have comparably small emission fluxes of up to 0.5 g m^−2^ at 09 UTC (Figure 18a–18b). In contrast, the dust emission in S3 is largest at 09 UTC throughout the year and reaches maximum values around 2 g m^−2^between January and March (Figure 18c). The diurnal cycle of dust emission in the S4 subdomain is similar to S3 but produces peaks of only up to 0.5–0.7 g m^−2^at 09 UTC (Figure 18d).

[72] The NLLJ breakdown appears generally more important for dust emission in the southern subdomains (Figure 18). The spatial mean contribution varies between 5% and 28%. More than 20% is found in S1 and S2 for April (Figure 18a–18b), in S3 for November–May (Figure 18c), and in S4 for November–April (Figure 18d). In S2, S3, and S4, maxima of the mineral dust emission flux at 09 UTC coincide well with peak contributions from the NLLJ breakdown.

[73] The phases of the annual cycles correspond well in S3 and S4, where clear regional NLLJ maxima are identified. At the same time, the mineral dust emission and the relative contribution to the total dust emission show clear mid-morning maxima at 09 UTC. This suggests NLLJs as an important driver for dust emission in the southeastern subdomains, which is in agreement with other studies [*Washington and Todd*, 2005; *Schepanski et al.*, 2009]. In contrast, the annual cycles of the contribution of NLLJs and dust emission in the S1 subdomain are not in phase. The driving meteorological processes here and for the northern subdomains will be discussed further in section 8.

## 8 Discussion

### 8.1 NLLJs as a Driver for Dust Emission

[74] The present study determined the dust emission amount associated with NLLJs. The simultaneous occurrence of NLLJs and dust emission is frequent in southeastern areas of North Africa including the Bodélé Depression, which is in agreement with the literature [*Washington and Todd*, 2005; *Schepanski et al.*, 2009]. S1, however, shows no agreement in the annual cycle of dust emission and NLLJ contribution but comparably large dust emission amounts between June and September. Here, the West African Monsoon system determines the meteorological conditions. Different peak-wind generating processes have been suggested, but the key drivers for dust emission remain controversial. The interaction of extratropical disturbances with African Easterly Waves has been proposed as a large-scale mechanism by *Knippertz and Todd* [2010]. On the mesoscale, the breakdown of NLLJs [e.g., *Schepanski et al.*, 2009] and cold pool outflows (haboobs) from deep moist convection [e.g., *Marsham et al.*, 2011] can cause dust emission. Relevant meteorological processes on smaller scales include rotating and nonrotating plumes in the convective BL [*Koch and Renno*, 2005]. The results of the present study suggest a 5–35% contribution from NLLJs along the margins of the Saharan heat low to West African dust emission during the monsoon season. Uncertainty due to the three hourly resolution that misses 09 LT remains in this area.

[75] NLLJs are not a major generator of dust emission for most source areas north of 25°N suggested by a relatively small NLLJ frequency in most months, a small total contribution, and a shift in the phases of the annual cycles for the dust emission fluxes and the NLLJ contribution. The small NLLJ contribution to dust emission north of 25°N is in agreement with the geographical distribution of DSA frequency during the morning from *Schepanski et al*. [2009]. An underestimation of the NLLJ contribution during the morning due to the weakening of jets by turbulence at night appears unlikely, as the dust emission amount is substantially larger at 12 UTC and 15 UTC when the NLLJ breakdown cannot be the driving mechanism. Other processes have to generate the dust-emitting peak winds in this case. Fronts associated to cyclones along the northern margins of the Sahara and in the Mediterranean region [e.g., *Alpert et al.*, 1990] are proposed as a dust-emitting process in the north during this time of year. The activity of Saharan cyclones peaks in northern hemisphere winter and spring [*Bou Karam et al.*, 2010], the time of year when the total dust emission in the northern subdomains is largest.

### 8.2 Implications for Dust Modeling

[76] Satellite observations suggest that dust emission peaks in the mid-morning and decreases in the afternoon [*Schepanski et al.*, 2009]. The morning peak has been associated with the breakdown of NLLJs. The dust emission maximum of NLLJs is expected around 09 LT, when the vertical mixing in the convective BL erodes the nighttime surface inversion over a certain depth. The six-hourly reanalysis data does not include this time since 06 UTC corresponds to about 05–08 LT and 12 UTC to 11–14 LT across North Africa. This has the following implications for dust emission modeling:

1. [77] Calculating offline dust emission with temporally coarse (several hours) resolution data may not simulate realistic results due to the importance of meteorological processes at intermediate times.
2. [78] Results for the total dust emission and the dust emission associated with NLLJs in North Africa are likely to be underestimated when the offline simulation is based on ECMWF reanalysis alone.

[79] The present study uses three-hourly data for dust emission simulations in North Africa, which fills the gap at 09 UTC (08–11 LT) to some extent. It has been shown that the DSA frequency and the amount of mineral dust emission increases in most North African regions in the mid-morning based on the ECMWF ERA-Interim forecasts. The satellite-based DSA frequency by *Schepanski et al.* [2009] documents less frequent dust emission events at night by a factor of 16–84 dependent on their defined region. The nocturnal DSA in the present study is only three times smaller than the DSA during the mid-morning. At the same time, the contribution of NLLJ momentum for 21–03 UTC in terms of dust emission amount is surprisingly large. The magnitude of dust emission due to nocturnal mixing is similar to the emission from the morning breakdown in four of seven subdomains. Whether the nighttime emissions are realistic, it needs to be assessed by comparing with long-term observational data. An estimate of the diurnal cycle of dust emission from observation is not given in the literature.

[80] Another way to investigate the plausibility of nighttime dust emission is a validation of the meteorological mechanism in the model. The validation of ERA-Interim under European conditions suggests an underestimation of the strengths of inversions and NLLJ wind speeds due to tuning of the physical parameterization scheme [*Sandu et al*., 2013]. A comparison of the wind speed profiles from ERA-Interim forecasts to radiosondes launched at Tombouctou, Agadez, and Niamey during the AMMA field campaign in 2006 [*Parker et al.*, 2008] shows that ERA-Interim produces NLLJs satisfactorily but underestimates the NLLJ strength and the vertical wind shear (section 4). This result is in line with the artificially increased values for the vertical mixing under stable stratification in the model [*Sandu et al*., 2013]. The finding implies that nocturnal turbulence in ERA-Interim and, therefore, the calculated dust emission with the offline dust model by *Tegen et al.*[2002] presented here might be overestimated at night. The diurnal cycle would be altered such that the mineral dust emission and the NLLJ contribution is underestimated during the mid-morning due to less available NLLJ momentum at sunrise. Quantifying a net effect on dust emission is, however, uncertain due to the nonlinear parameterization of dust emission as a function of the 10 m wind speed. A validation of the NLLJ in ERA-Interim with a long-term observational data set over North Africa would be necessary to determine the uncertainty, but such data is not available. The effect of nocturnal turbulence on the diurnal cycle of dust emission north of 25°N is, however, expected to be negligible in most months and source areas as other meteorological processes appear to generate dust-emitting peak winds more efficiently (section 8.1).

### 8.3 Limitations

[81] Limitations of the present work arise from the relatively coarse temporal and spatial resolution as well as the physical parameterizations of both dust emission and the near-surface wind speed in the nocturnal BL. A validation of NLLJs in ERA-Interim against a few months of observation indicates an underestimation of the upper end of the distribution for the core wind speed and height (section 4). These NLLJs are the driver for generating peak winds for dust emission (section 6.3). The nonlinearity, however, inhibits a conclusion whether the associated dust emission is overestimated or underestimated.

[82] Further uncertainties are due to the sensitivity of the detection algorithm to the chosen threshold values for the strengths of the surface inversion and the vertical wind shear above the jet core (section 3). It is essential to acknowledge the chosen NLLJ definition for interpreting the presented results. The robustness of the NLLJ contribution to dust emission shall be evaluated with a sensitivity test of the detection algorithm applied to the ERA-Interim forecasts in the following. Although a vertically narrow layer of high wind speed has been defined as a criterion for the NLLJ definition, the shape of the NLLJ is not necessarily relevant for the associated dust emission. Especially under relatively strong large-scale forcing, NLLJs may not show a strong wind speed decrease above the NLLJ core. In order to test how the shape influences the NLLJ contribution to mineral dust emission, the vertical wind shear criterion is switched off. The result shows that the spatial mean contribution of NLLJ structures to dust emission increases by a factor of 2 to 3 due to more identified jets in the test. The annual cycle and the relative importance of different times of the days, however, are not substantially affected. This finding gives confidence in the presented results for the NLLJ contribution to dust emission, but the absolute amount of NLLJ contribution to dust emission needs to be treated with caution.

## 9 Conclusion

[83] In this study, a quantitative NLLJ climatology was produced for North Africa, based on ECWMF ERA-Interim data for 1979–2010. Detailed characteristics of the wind speed maxima and the contribution of the downward mixing of their momentum to mineral dust emission were analyzed in a climatological sense. The work is based on a newly developed automatic NLLJ detection algorithm. A wind speed maximum between the lowest model level and 1500 m agl is detected as a NLLJ, if the surface layer is stably stratified and the vertical wind shear above the jet core exceeds a certain threshold.

[84] The results emphasize a frequent NLLJ occurrence of 29% in the annual and spatial mean over North Africa. NLLJ frequencies of up to 80% are found in NLLJ hot spots. Here, low-level baroclinicity and orographic channeling are suggested to favor their formation. Baroclinicity has been identified as favorable condition for the NLLJ hot spots along the margins of the heat low between April and September. A NLLJ hot spot due to mountain channelling is the Bodélé Depression between November and March. Typical median heights and wind speeds of NLLJs in ERA-Interim are 350 m agl and 10 m s^−1^, respectively.

[85] NLLJs are a source of momentum for mineral dust emission in North Africa. The downward mixing of NLLJ momentum by nocturnal turbulence and the morning breakdown is associated with 15% of North African dust emission annually and spatially averaged. Up to 60% of the total dust emission can be associated with NLLJs, dependent on the region and the time of year. Breakdowns of the NLLJ are particularly important for dust emission in the southeast of North Africa at the beginning of the year. The peak contributions of NLLJs to mineral dust emission around 09 LT over North Africa underline the importance of using wind speed data of sufficient temporal resolution in a dust emission model. Six-hourly wind speeds do not capture the mid-morning maximum over North Africa. Uncertainties of the results remain due to the sensitivity of the NLLJ detection algorithm to the threshold values, physical parameterizations, temporal, and spatial resolution.

[86] Other meteorological processes than the vertical mixing of NLLJ momentum appear to be relatively more important for dust emission west of 10°E and generally north of 25°N. The main driving meteorological condition for dust emission in the western Sahara remains controversial, but the present work suggests seasonal mean NLLJ contributions to the mineral dust emission of 5–35%. Extra-tropical cyclones are suggested as driving mechanisms for dust emission along the northern margins of the Sahara desert that will be addressed in future work.

[87] The new automated detection algorithm for NLLJs presented here will be used for evaluating the wind speed maxima and associated dust emission in weather and climate models. It holds the potential for further improving the simulations of the diurnal cycle and the total amount of mineral dust emission.

## Key Points

1. Long-term climatology of Nocturnal Low-Level Jets (NLLJ) over North Africa
2. Newly developed automated detection algorithm for NLLJs
3. NLLJs contribute 15 % to North African dust emission in annual and spatial mean
